# Effects of Dietary Wild Thyme Alone or Combined with Probiotic or Prebiotic Supplementation on Growth Performance, Immune Response, Oxidative Status, and Intestinal Health in Broiler Chickens

**DOI:** 10.3390/vetsci13080785

**Published:** 2026-08-05

**Authors:** Ahmad A. Aboragah, Abdulrahman S. Alharthi, Hani H. Al-Baadani

**Affiliations:** Department of Animal Production, College of Food and Agriculture Sciences, King Saud University, Riyadh 11451, Saudi Arabia; aaboragah@ksu.edu.sa (A.A.A.); abalharthi@ksu.edu.sa (A.S.A.)

**Keywords:** *Thymus vulgaris*, biotic supplementation, intestinal integrity, microbiota, broiler performance

## Abstract

(1) Background: The global transition away from antibiotic growth promoters (AGPs) requires effective, sustainable alternatives to maintain broiler performance and intestinal homeostasis. This study evaluated the effects of dietary wild thyme powder, administered alone or combined with a prebiotic or probiotic, as potential AGP alternatives on growth performance, serum oxidative status, immune response, cecal microbiota composition, short-chain fatty acid (SCFA) production, ileal histomorphometry, and intestinal barrier integrity in broilers. (2) Methods: Five hundred forty-six male Ross 308 chicks were assigned to seven treatments (n = 13 replicates/group) for 28 d: T1 (basal diet); T2 (0.5 g/kg antibiotic Neoxyval); T3 (1.0 g/kg thyme); T4 (0.75 g/kg prebiotic); T5 (0.50 g/kg probiotic); T6 (prebiotic + thyme); and T7 (probiotic + thyme). Growth performance was measured, and one bird from each replicate was randomly sampled at 28 d for multi-endpoint biological analyses. (3) Results: Birds that received T3 to T7 had increased final body weight and weight gain. Cumulative feed conversion ratio was improved in the T3, T5, and T7 groups, with T7 displaying numerical superiority over individual additive groups without reaching statistical synergy. All-natural supplements (T3, T5, T6, T7) lowered serum malondialdehyde and enhanced interferon-γ. Birds receiving T6 showed increased tumor necrosis factor-α, whereas birds receiving T3 and T4 showed reduced levels. Probiotic-inclusive diets (T5 and T7) significantly increased cecal propionic acid, total SCFAs, and ileal villus surface area. Natural additive treatments broadly promoted cecal eubiosis by increasing *Lactobacillus* spp. while decreasing *Clostridium* spp. Crucially, combining thyme with prebiotics (T6) or probiotics (T7) synergistically upregulated ileal tissue occludin concentrations. (4) In conclusion, under the conditions of this trial, dietary supplementation with wild thyme powder, a MOS/β-glucan prebiotic, and a *Bacillus licheniformis* probiotic, particularly the thyme + probiotic combination, was an effective regimen for supporting growth performance, cecal eubiosis, and ileal barrier integrity in healthy broilers. However, these findings are limited to a single dose level (1.0 g/kg thyme), a single plant source, and non-challenged conditions; further research incorporating dose–response evaluations, disease-challenge models, and diverse phytogenic origins is necessary to confirm broader field applicability.

## Simple Summary

As the commercial poultry industry transitions away from antibiotic growth promoters, farm managers and breeding practitioners need reliable, natural feeding strategies that maintain flock growth, feed efficiency, and intestinal robustness. This study examined whether blending wild thyme powder with standard prebiotics or probiotics could replace conventional antibiotics in broiler diets over a 28-day grow-out period. Our findings show that adding thyme powder, especially when paired with a spore-forming prebiotic, significantly increases final bird weight and improves overall feed conversion efficiency to levels that match or exceed antibiotic treatment. At the tissue level, these natural combinations act as a dual protective shield: thyme and beneficial microbes strengthen the intestinal lining, promote friendly gut bacteria, and enhance nutrient absorption. For poultry producers, combining thyme powder (1.0 g/kg) with a probiotic (0.50 g/kg) offers a viable, cost-effective, non-antibiotic strategy to optimize flock productivity, safeguard gut health, and reduce production losses in modern antibiotic-free broiler operations.

## 1. Introduction

The global poultry industry operates under intense pressure to optimize growth performance and feed efficiency while ensuring bird health and welfare [1]. For decades, sub-therapeutic doses of AGPs were routinely incorporated into broiler diets to stabilize the intestinal microbiota, mitigate subclinical inflammation, and enhance growth indices [2]. Although AGPs delivered measurable production benefits, their prolonged use has accelerated the emergence of antibiotic resistant bacteria, raising urgent global concerns for both animal and public health [3]. Consequently, regulatory bodies worldwide have restricted or banned AGP use in poultry production [4]. In regions such as the Kingdom of Saudi Arabia, where commercial broilers are rapidly reared within a compact 28-day production cycle, identifying non-antibiotic alternatives is crucial [5]. Removing AGPs often triggers dysbiosis, compromised mucosal barrier integrity, and oxidative stress, driving the search for effective natural alternatives [6].

Phytogenic additives and biotics are promising AGP alternatives [7,8]. Wild thyme (*Thymus vulgaris* L.) has gained substantial attention due to its high concentration of volatile essential oils, primarily the phenolic monoterpenes thymol and carvacrol [9]. These compounds exhibit potent antimicrobial activity by disrupting bacterial cell membranes and possess exceptional radical-scavenging capabilities that bolster systemic antioxidant defenses, thereby improving the intestinal ecosystem and nutrient utilization [10]. Concurrently, biotic supplements (prebiotics and probiotics) have emerged as reliable tools for gut health optimization [11]. For example, mannan-oligosaccharide (MOS)/β-glucan prebiotics bind enteric pathogens and modulate gut-associated immunity [12], while spore-forming probiotics such as *Bacillus licheniformis* promote competitive exclusion and epithelial barrier repair [13].

Several studies have confirmed that dietary thyme powder improves broiler growth performance, including weight gain and feed conversion ratio, enhances serum antioxidant capacity, strengthens humoral immunity, favorably modulates cecal microbiota composition, and improves ileal villus morphometry [14,15]. MOS and β-glucans reinforce colonization resistance, improve gut morphology, and reduce pathogen load in a manner that is mechanistically complementary to the direct antimicrobial action of phytogenic compounds [16,17]. *Bacillus licheniformis*, as a probiotic, promotes intestinal homeostasis by competitively excluding enteric pathogens, upregulating tight junction protein expression (e.g., occludin), stimulating anti-inflammatory cytokine profiles, and modulating the intestinal microbiome toward a *Lactobacillus*- and *Bifidobacterium*-enriched community, thus leading to measurable improvements in growth performance, feed efficiency, intestinal morphology, and antioxidant status in broiler chickens [18,19]. Although these individual additives consistently support growth performance, antioxidant balance, cecal eubiosis, and mucosal structure, their combined applications remain insufficiently explored.

The concept that phytogenic antibacterial activity, paired with prebiotic-mediated pathogen exclusion and probiotic-driven colonization enhancement, may generate combined effects beyond single-agent supplementation has been proposed but rarely tested using comprehensive, multi-endpoint designs [20]. Recent studies have reported performance and histological improvements when combining phytogenics with prebiotics [21] or probiotics [22]. However, a study simultaneously evaluating both thyme + prebiotic and thyme + probiotic combinations against the individual components and an antibiotic reference, while comprehensively assessing growth performance, oxidative status, cytokine profiles, intestinal tight junction markers (occludin), cecal microbiota, SCFAs, and ileal histomorphology, has not been reported.

We hypothesized that dietary wild thyme powder, alone or combined with a prebiotic or probiotic, would improve growth performance, antioxidant status, immune response, gut microbiota, and intestinal health in broiler chickens. Therefore, the objective of this study was to evaluate the effects of these dietary strategies on growth performance, oxidative status, inflammatory markers, cecal microbiota, SCFA production, and intestinal integrity during a 28-day production cycle.

## 2. Materials and Methods

### 2.1. Ethical Statement

All experimental procedures involving birds, including blood collection and tissue sampling, received prior ethical approval from the Research Ethics Committee of King Saud University (approval no. KSU-SE-21-47) before any animals were enrolled in the study.

### 2.2. Birds and Trial Design

Five hundred and forty-six Ross 308 male chicks were obtained from a commercial hatchery in Riyadh, Saudi Arabia. All chicks were individually weighed on arrival (day 0), and sex was determined by feather characteristics. Birds were randomly assigned to one of seven treatment groups (78 birds per group) using a completely randomized design. The trial lasted 28 d, matching the standard commercial slaughter age in Saudi Arabia. Within each group, 13 replicate cages were used (n = 6 birds per cage), each measuring 0.5 m^2^. This setup was designed to achieve a target final stocking density of approximately 20 kg/m^2^ by day 28, based on anticipated commercial market weights, ensuring compliance with local animal welfare standards while mimicking intensive production conditions.

Housing conditions during the trial followed Aviagen Ross 308 management guidelines (Aviagen, 2018, New York, NY, USA). The brooding temperature was set at 35 °C on day 0 and gradually decreased to 19 °C by day 21, while relative humidity was reduced from 55% to 40% over the same period. During the first seven days, birds received continuous lighting at 35 lux. From day 8 onward, the lighting schedule was 18 h of light at 10 lux followed by 6 h of darkness [7,8]. All chicks received hatchery-administered spray vaccinations against Newcastle disease, infectious bursal disease, and infectious bronchitis (Fort Dodge, IA, USA).

### 2.3. Additive Sourcing and Characterization

Neoxyval, a commercial preparation containing 200 mg oxytetracycline and 200 mg neomycin, was obtained from Kela Co., Ltd., Hoogstraten, Belgium. Thyme powder (*Thymus vulgaris* L.) was collected as whole plants from the valleys of Ibb City, Yemen. The prebiotic supplement (a yeast cell wall complex derived from Saccharomyces cerevisiae, containing 16.12% MOS and 17.95% β-glucan) was obtained from Biochem, Lohne, Germany. The probiotic preparation, consisting of viable endospores of *Bacillus licheniformis* (strain DSM 5749) with a guaranteed minimum concentration of 3.2 × 10^9^ colony-forming units (CFUs) per gram of powder, was obtained from Huvepharma, Sofia, Bulgaria.

### 2.4. Experimental Diets

Throughout the 28-day trial, birds received a two-phase mash diet formulated to meet the nutritional requirements of the Ross 308 strain at each production stage (Aviagen, 2022, New York, NY, USA): a starter phase from days 0 to 14 and a grower phase from days 15 to 28. Yellow corn, soybean meal, and soybean oil were the main dietary ingredients, with the complete composition and calculated nutrient content shown in Table 1.

Seven treatment groups were established. The control group (T1) received the unsupplemented basal diet throughout the experiment. An antibiotic reference group (T2) served as a positive control, with the basal diet supplemented with 0.5 g/kg Neoxyval (oxytetracycline and neomycin). The remaining five treatments evaluated natural feed additives as potential antibiotic alternatives. Birds in T3 received thyme powder (*Thymus vulgaris* L.) at 1 g/kg diet. T4 and T5 tested a prebiotic and a probiotic individually: the MOS/β-glucans prebiotic (0.75 g/kg) and the *Bacillus licheniformis* probiotic (0.50 g/kg). The final two groups assessed whether combining thyme with a biotic supplement produced additive benefits: T6 received the prebiotic (0.75 g/kg) together with thyme powder (1 g/kg), and T7 received the probiotic (0.50 g/kg) together with thyme powder (1 g/kg). The specified inclusion doses for all additives (antibiotic, thyme, prebiotic, and probiotic) were kept at the same fixed rates in both the starter (0–14 d) and grower (15–28 d) dietary phases.

All commercial feed additive inclusion rates (antibiotic, prebiotic, and probiotic) were implemented in strict accordance with manufacturer recommendations. Meanwhile, the thyme powder inclusion level was selected based on our laboratory chemical analysis, preliminary in-house trials, and previous literature demonstrating its optimal efficacy for broiler growth performance and physiological traits [23].

Quantitative characterization of wild thyme powder across three representative batches (n = 3), determined by high-performance liquid chromatography (HPLC) and gas chromatography–mass spectrometry (GC-MS) following standard analytical procedures [24,25], as shown in Table 2, revealed total phenolics of 38.45 ± 1.20 mg gallic acid equivalents (GAE)/g and total flavonoids of 14.12 ± 0.85 mg quercetin equivalents (QE)/g. The major bioactive constituents were thymol (72.92 ± 1.45%; 38.25 ± 0.95 mg/g DM powder), carvacrol (2.16 ± 0.12%; 1.13 ± 0.08 mg/g DM powder), *p*-cymene (4.85 ± 0.22%; 2.54 ± 0.11 mg/g DM powder), and gamma-terpinene (3.12 ± 0.18%; 1.63 ± 0.09 mg/g DM powder). At a 1.0 g/kg inclusion level in treatments T3, T6, and T7, the finished diets provided delivered doses of 38.25 mg/kg diet thymol and 1.13 mg/kg diet carvacrol, supplying an estimated daily intake of 1.25 mg thymol/kg BW/day and 0.037 mg carvacrol/kg BW/day during the starter phase (0–14 d), and 1.38 mg thymol/kg BW/day and 0.041 mg carvacrol/kg BW/day during the grower phase (15–28 d).

### 2.5. Growth Performance Measurements

Body weight of birds was recorded individually for each replicate (cage) on the day of arrival (0 d, initial weight) and at the end of each dietary phase: 14 d for the starter period and 28 d for the grower period. Weight gain for each phase and for the entire 28-day trial was calculated by subtracting the initial body weight from the final body weight within each measurement interval, as described by Golshahi et al. [22]. Feed consumption was monitored on the same two-week schedule by weighing the feed offered and subtracting unconsumed residues at the end of each phase (0–14 and 15–28 d), as well as cumulatively for the entire trial (0–28 d). These records were used to calculate feed conversion ratio, defined as the quantity of feed consumed per unit of live weight gained, as described by Al-baadani et al. [7]. The cage was used as the unit of measurement for all growth performance data.

### 2.6. Serum Oxidative Status and Immune Response

At 28 d, one bird per cage was randomly selected for blood collection, yielding 13 samples per dietary treatment. Each bird was manually restrained, and 2 mL of blood was drawn from the wing vein into plain tubes (without anticoagulant). Serum was obtained by centrifuging the clotted samples at 3000× *g* for 20 min using a Hettich Zentrifugen Mikro 200R centrifuge (Tuttlingen, Germany). The recovered serum was stored at −80 °C until the day of assay.

Two oxidative stress biomarkers were quantified in serum: total antioxidant capacity (TAC), reflecting overall non-enzymatic antioxidant defense, and radical-scavenging potential across serum constituents, and malondialdehyde (MDA), a well-established end-product of lipid peroxidation used as an index of cellular oxidative damage. Both were measured with commercially available ELISA kits (Solarbio Life Sciences, Beijing, China) and read on a microplate reader (Bio-Medical Electronics, Shenzhen, China), strictly following the manufacturer’s step-by-step protocols. The TAC assay was based on the ferric-reducing antioxidant power method, in which serum antioxidants reduce ferric ions (Fe^3+^) to ferrous ions (Fe^2+^), forming a colored complex measured at 593 nm. Serum MDA was determined using the thiobarbituric acid (TBA) reaction, in which MDA reacts with TBA at high temperature (95 °C) under acidic conditions to yield a pink chromogenic MDA–TBA complex measured at 532 nm. These two indicators were selected to provide an integrative operational assessment of systemic oxidative balance and overall tissue protection.

Inflammatory immune markers including interleukin-6 (IL-6), tumor necrosis factor-α (TNF-α), and interferon-γ (IFN-γ), were quantified from the same serum aliquots using species-specific ELISA kits (Bioassay Technology Laboratory, Jiaxing, China) and an MR-96A microplate reader (Mindray Bio-Medical Electronics Co., Ltd., Shenzhen, China), following the procedure reported by Xiao et al. [18].

### 2.7. Quantification of Selected Cecal Microbiota by RT-qPCR

At 28 d, cecal digesta (1 g) was aseptically collected from one bird per cage into sterile cryotubes (13 samples per dietary treatment in total). Samples were immediately immersed in liquid nitrogen and subsequently stored at −80 °C until DNA extraction. Total genomic DNA was isolated from each sample using the QIAamp DNA Stool Mini Kit (Qiagen, Germantown, MD, USA), with all steps performed in strict accordance with the manufacturer’s protocol. Extracted DNA was assessed for yield and purity using a NanoDrop 2000 spectrophotometer (Thermo Fisher Scientific, Waltham, MA, USA) by recording absorbance at 260 nm (A260) and the A260/A280 ratio. All samples were then normalized to a working concentration of 100 ng/µL prior to amplification.

Quantitative real-time PCR was performed using an Applied Biosystems 7300 Real-Time PCR System (Thermo Fisher Scientific, Waltham, MA, USA) with SYBR Green PCR Master Mix (Applied Biosystems, Thermo Fisher Scientific, Waltham, MA, USA). Five bacterial taxa, *Lactobacillus* spp., *Bifidobacterium* spp., *Bacteroides* spp., *Clostridium* spp., and *Escherichia coli*, were selected (Table 3) as targeted biological indicators because they represent core functional categories of avian cecal eubiosis, spanning dominant beneficial saccharolytic/lactogenic commensals, primary propionate-producing fermenters, and major opportunistic or pathogen-associated groups that are sensitive to phytogenic phenolics and biotic exclusion. Targeted qPCR was intentionally selected over 16S rRNA amplicon sequencing to achieve absolute quantification, expressed as log_10_ gene copy numbers per gram of digesta. To ensure reproducibility, each sample–primer combination was run in triplicate. To achieve rigorous absolute quantification in compliance with MIQE guidelines, individual taxon-specific standard curves were generated for each bacterial target using 10-fold serial dilutions (10^8^ to 10^2^ gene copies per reaction) of purified recombinant plasmid vectors (pGEM-T Easy Vector System, Promega, Madison, WI, USA) containing verified 16S rRNA gene inserts of each respective bacterial species [26]. Amplification efficiencies (E) were calculated from the standard curve slopes using the formula E = [10 ^(−1/slope)^ − 1] × 100%. Assay performance metrics for all five target taxa met stringent quality thresholds: amplification efficiencies ranged from 94.1% to 103.5%, coefficients of determination (R^2^) exceeded 0.994, and limits of detection were ≤ 50 gene copies per reaction. Specificity of each reaction product was verified post-cycling by thermal melt-curve analysis (65 to 95 °C with increments of 0.5 °C every 5 s), confirming a single, distinct dissociation peak for each target without nonspecific primer-dimer artifacts, and by validation with 2.0% agarose gel electrophoresis. Bacterial abundances are reported as log_10_ gene copy numbers per gram of cecal digesta.

**Table 3:** Oligonucleotide primer sequences used for real-time PCR quantification of selected cecal microbiota.

Target Gene	Primer Sequences (5′→3′)	GenBank Accession Number
*Lactobacillus* spp.	F: CGACTGCTCTGGTTATACCGT R: TGAAGAAGGGTTTCGGCTCG	NR_040806.1
*Bifidobacterium* spp.	F: CAGCTCGTGTCGTGAGATGT R: GATCTGACGTCATCCCCACC	NR_043433.1
*Bacteroides* spp.	F: TAGAGATAAGGCCCTTGGGGT R: CGAATCGGAGTATTTAGGTGC	NR_029219.1
*Clostridium* spp.	F: GTTTACGGCGTGGACTACCA R: TGGAAGTCTAGAGTGCGGGA	NR_114788.1
*Escherichia coli*	F: CATGCCGCGTGTATGAAGA R: GGGTAACGTCAATGACAAAG	NR_024570.1

Primers were designed using Primer-BLAST (NCBI) (https://www.ncbi.nlm.nih.gov/tools/primer-blast/, accessed on 2 July 2026) to ensure target specificity and prevent cross-reactivity with non-target sequences.

### 2.8. Cecal SCFA Quantification

Cecal digesta collection for SCFA analysis followed the same protocol used for microbiota sampling: 1 g of digesta was aseptically obtained from one bird per replicate (13 birds per dietary treatment), immediately transferred into sterile cryotubes, snap-frozen in liquid nitrogen, and stored at −80 °C until analysis. Individual and total SCFA concentrations—acetic, propionic, and butyric acids—were determined by gas chromatography–mass spectrometry (GC-MS; Agilent Technologies, 1260 Series, Palo Alto, CA, USA), using an authenticated mixture of the same three acids as internal standards (Augsburg, Germany). The analytical procedure followed methods previously validated [27]. Results for each acid and for total SCFAs are expressed as milligrams per gram of cecal digesta.

### 2.9. Ileal Histomorphology

Intestinal morphometry samples were collected at 28 d, with one bird per replicate selected for tissue collection (13 birds per dietary treatment). A 2 cm segment was excised from the mid-ileum, taking care not to stretch or compress the tissue wall, as mechanical distortion introduces measurement error in villus dimensions. Segments were fixed in 10% phosphate-buffered formalin for 72 h, then dehydrated through an ascending ethanol series using a Tissue-Tek VIP 5 Jr automated tissue processor (Sakura, Tokyo, Japan). Each sample was individually embedded in paraffin wax at 60 °C for 60 min and sectioned on a rotary microtome (Leica Biosystems, Nussloch, Germany) (one section per bird), preserving the cage-as-replicate structure of the experimental design.

Sections were stained using the standard hematoxylin and eosin protocol, with hematoxylin labeling cell nuclei and eosin counterstaining the cytoplasm and extracellular matrix. Morphometric measurements were taken on six well-oriented villi per section under a Micros MC500 microscope (Micros Austria, Hunnenbrunn, Austria) equipped with an AmScope digital camera and image analysis software (Version 3.7, AmScope, Irvine, CA, USA), yielding villus length, villus width, and crypt depth for each replicate [28]. Two derived indices were calculated: the villus length-to-crypt depth ratio, obtained by dividing villus length by crypt depth, and villus surface area, computed as 2π × (villus width ÷ 2) × villus length [22].

### 2.10. Ileal Tissue Occludin as an Intestinal Integrity Marker

At 28 days of age, a one-cm segment of ileal tissue was collected from one bird per cage (13 birds per treatment group) immediately after slaughter, adjacent to the sections harvested for histomorphological analysis, and snap-frozen in liquid nitrogen before storage at −80 °C until processing. Ileal tissue samples (25 mg) were homogenized in 250 µL of ice-cold phosphate-buffered saline (PBS, 10 mM, pH 7.4) using a handheld tissue homogenizer. Homogenates were centrifuged at 3000× *g* for 10 min at 4 °C, and the resulting supernatants were collected and used immediately for analysis. The concentration of occludin, a transmembrane tight junction protein and key determinant of intestinal epithelial barrier integrity, was quantified in ileal tissue homogenates using a commercially available chicken-specific enzyme-linked immunosorbent assay (ELISA) kit (Bioassay Technology Laboratory, Jiaxing, China), according to the manufacturer’s instructions. Absorbance was measured at 450 nm using a microplate reader (MR-96A; Mindray Bio-Medical Electronics Co., Ltd., Shenzhen, China). Occludin concentrations were expressed as nanograms per milligram of total protein (ng/mg protein), with total protein content determined by the Bradford assay to allow normalization across samples. Occludin was selected as the primary barrier marker because it is a critical transmembrane regulator of inter-epithelial tight junction stability and macromolecular permeability, serving as an effective proxy for physical mucosal barrier robustness under non-challenged feeding conditions, although other structural tight junction components were not assessed.

### 2.11. Statistical Analysis

The definition of the experimental unit varied by response variable: the replicate cage (n = 13 per treatment) was defined as the experimental unit for all performance traits. For all biological endpoints, including oxidative status, serum cytokines, qPCR microbiota counts, SCFA concentrations, ileal histomorphometry, and occludin levels, one representative bird per cage was randomly selected and sampled at 28 d (n = 13 sampled birds per treatment). Because each sampled bird originated from a distinct, independent cage replicate, a strict 1:1 mapping between sampled individuals and cage replicates was maintained. Prior to initiating the study, an a priori statistical power analysis was performed using G*Power 3.1. Based on historical variance estimates from comparable broiler nutrition studies evaluating growth performance and gut health parameters, a medium-to-large effect size (f = 0.35) was specified, ensuring statistical power exceeding 85% to detect meaningful treatment differences. Before the main analysis, datasets were screened for normality and homogeneity of variances using the Shapiro–Wilk and Levene’s tests to ensure that parametric assumptions were met.

All data were analyzed as completely randomized design using the General Linear Model procedure of SAS Institute, version 9.4 [29]. Differences among individual treatment means were separated using Tukey’s honest significant difference (HSD) test at *p* < 0.05. In addition, data were analyzed using a two-step strategy that combined a 2 × 2 × 2 factorial arrangement (evaluating treatments T3–T7) with planned single-degree-of-freedom orthogonal contrasts comparing natural additives with controls. Factorial main effects (T1 × T3, T1 × T4, and T1 × T5) and their two-way and three-way interaction terms (T3 × T4, T3 × T5, T4 × T5, and T3 × T4 × T5) were evaluated to assess synergy and additivity. To assess comparisons involving the control (T1) and antibiotic (T2), two a priori orthogonal single-degree-of-freedom contrasts were defined (T1 vs. all-natural additives [T3–T7] and T2 vs. all-natural additives [T3–T7]). All results are presented as treatment means with the standard error of the mean (SEM).

## 3. Results

### 3.1. Growth Performance

The productive performance of broiler chickens in response to treatment groups receiving thyme powder, a prebiotic, a probiotic, and their combinations is shown in Table 4. No significant treatment effects (*p* > 0.05) were detected for any performance parameter during the starter phase (0–14 d). Birds receiving T3, T4, T5, and T7 had higher final body weight (28 d) and weight gain (15–28 d or 0–28 d) than T1 (*p* < 0.05), but did not differ significantly from T2 or T6. Feed intake was unaffected by the treatments throughout the trial (*p* > 0.05). In addition, feed conversion ratio was unaffected by treatment during the starter phase (*p* > 0.05). In contrast, feed conversion ratio was significantly improved in birds receiving T7 during the grower phase (15–28 d) compared with T1 (*p* = 0.014). Over the full 28 d trial, a significant overall treatment effect was observed for cumulative feed conversion ratio (ANOVA, *p* = 0.001). Tukey’s post hoc test showed that birds receiving T3, T5, and T7 achieved significantly lower feed conversion ratio values than the control T1 group (*p* < 0.05), while remaining statistically comparable to T2, T4, and T6.

The contrasts comparing natural additives with controls (T1 or T2) and thyme (T1 × T3), prebiotic (T1 × T4), and probiotic (T1 × T5) significantly affected 28-day body weight, overall weight gain (0–28 d), and feed conversion ratio during both the grower (15–28 d) and overall periods (*p* < 0.05). In contrast, no significant two-way or three-way interaction effects were observed for any growth performance parameter (*p* > 0.05).

### 3.2. Serum Oxidative Status

The serum oxidative status biomarkers of broiler chickens in response to treatment with thyme powder, prebiotic, probiotic, and their combinations are shown in Table 5. Birds receiving T3 and T7 had higher TAC concentrations than the other treatments (*p* = 0.004), except for the T5 group, which did not differ from the T3 and T7 groups. The malondialdehyde (MDA) concentration in serum was reduced in T3, T5, T6, and T7 compared with birds in the T1, T2, and T4 groups (*p* = 0.001). The contrasts comparing natural additives with controls (T1 or T2) and thyme (T1 × T3) or probiotic (T1 × T5) significantly affected TAC and MDA levels (*p* < 0.05), whereas the prebiotic (T1 × T4) showed no significant main effect (*p* > 0.05). Significant two-way interactions occurred between prebiotic and thyme (T3 × T4) for both TAC and MDA, as well as between prebiotic and probiotic (T4 × T5) for MDA (*p* < 0.05). Additionally, a significant three-way interaction (T3 × T4 × T5) was observed for TAC (*p* = 0.003) and MDA (*p* = 0.009), indicating substantial synergistic modulation among all three feed additives.

### 3.3. Immune Response Markers

The immune response markers of broiler chickens in response to treatment with thyme powder, prebiotic, probiotic, and their combinations are shown in Table 6. Serum IL-6 concentration increased in birds in the T3, T5, and T7 groups compared with the other treatment groups (*p* = 0.021). Birds receiving T6 showed increased serum TNF-α concentration, while birds receiving T3 and T4 showed reduced concentrations compared with the T1 and T2 groups (*p* = 0.001). Interferon-γ (IFN-γ) concentration was increased in the T3, T5, T6, and T7 groups compared with the T1 and T2 groups (*p* = 0.001). The contrast effect comparing natural additives with controls (T1 or T2) influenced IL-6 and IFN-γ levels (*p* < 0.05), while TNF-α was not affected (*p* > 0.05). The main effects of thyme (T1 × T3) and probiotic (T1 × T5) affected serum IL-6 and IFN-γ, with thyme also influencing TNF-α (*p* = 0.005), while the prebiotic (T1 × T4) showed no significant main effects on cytokine levels. Two-way interactions significantly altered IFN-γ in T3 × T4 and T4 × T5 combinations (*p* < 0.05), and TNF-α in the T3 × T5 combination (*p* = 0.001). Furthermore, a significant three-way interaction (T3 × T4 × T5) was confirmed for TNF-α (*p* = 0.003) and IFN-γ (*p* = 0.004), whereas serum IL-6 showed no significant interactions (*p* > 0.05).

### 3.4. Cecal SCFAs Concentrations

The cecal SCFA concentrations of broiler chickens in response to treatment with thyme powder, prebiotic, probiotic, and their combinations are shown in Table 7. All four SCFA fractions were significantly affected by treatment groups (*p* = 0.001). For cecal acetic acid concentration, treatment groups T4–T7 showed higher levels (*p* = 0.001) than the T1, T2, and T3 groups, which did not differ significantly from one another. Two probiotic-containing groups (T5 and T7) showed the highest propionic acid concentrations compared with the other treatment groups (*p* = 0.001). In addition, birds receiving T2 and T3 had higher concentrations than the T1, T4, and T6 groups. For cecal butyric acid, birds receiving T5 showed the highest concentration compared with the T1, T2, and T7 groups (*p* = 0.001). The total SCFA concentration was higher in birds receiving T5 and T7 than in the other treatment groups (*p* = 0.001). In addition, birds receiving T4 and T6 had higher concentrations than T1 and T2 groups but did not differ significantly from the T3 group.

The contrast effect comparing natural additives with controls (T1 or T2) influenced acetic acid and total SCFAs (*p* < 0.05), while propionic acid was affected in T1 vs. T3–T7 and butyric acid in T2 vs. T3–T7 (*p* = 0.001). Probiotic (T1 × T5) showed broad main effects across all SCFA fractions (*p* = 0.001). Thyme (T1 × T3) affected propionic acid alone, while the prebiotic (T1 × T4) affected acetic acid and total SCFAs (*p* < 0.05). Two-way interaction terms were highly significant, including T3 × T4 for acetic, propionic, and total SCFAs; T3 × T5 for all SCFA measures; and T4 × T5 for propionic, butyric, and total SCFAs (*p* < 0.05). A significant three-way interaction (T3 × T4 × T5) was also confirmed for acetic acid and total SCFAs (*p* = 0.001).

### 3.5. Cecal Microbiota Quantification

The cecal microbiota quantification in broiler chickens in response to treatment with thyme powder, a prebiotic, a probiotic, and their combinations is shown in Table 8. The treatment groups significantly influenced cecal counts of *Lactobacillus* spp., *Bifidobacteria* spp., *Clostridium* spp., and *Escherichia coli* (*p* < 0.05), whereas *Bacteroides* spp. counts were not significantly affected by any treatment (*p* > 0.05). Cecal *Lactobacillus* spp. counts were higher in birds receiving the T4–T7 treatments, followed by the T3 treatment, compared with birds in the T1 and T2 groups (*p* = 0.001). For cecal *Bifidobacteria* spp., birds receiving T7 showed higher counts than those in the T1 and T2 groups (*p* = 0.003), but did not differ significantly from the other treatment groups. *Clostridium* spp. counts were lower in birds receiving T3–T7 compared with those in the T1 group (*p* = 0.004). In contrast, *Clostridium* spp. counts in birds receiving the antibiotic reference (T2) were not significantly different from those in T1 or from the natural additive groups (T3–T7). Birds receiving T3 and T7 had lower *Escherichia coli* counts than those in T1, but did not differ significantly from the other groups (*p* = 0.003).

*Lactobacillus* spp. and *Bifidobacteria* spp. counts were affected in contrast tests comparing natural additives with controls, while *Bacteroides* spp. in T2 vs. T3–T7 and *Clostridium* spp. or *Escherichia coli* in T1 vs. T3–T7 differed significantly (*p* < 0.05). Cecal microbial populations responded significantly to individual additives and their interaction effects. The main effects of thyme (T1 × T3), prebiotic (T1 × T4), and probiotic (T1 × T5) all significantly affected *Lactobacillus* spp. and *Clostridium* spp., with probiotic addition also affecting *Bifidobacteria* spp., and thyme affecting *Bacteroides* spp. and *Escherichia coli* (*p* < 0.05). Two-way interactions significantly impacted *Lactobacillus* spp. and *Escherichia coli* (T3 × T4), as well as *Bacteroides* spp. and *Escherichia coli* (T3 × T5). A significant three-way interaction (T3 × T4 × T5) was observed for *Escherichia coli* (*p* = 0.007).

### 3.6. Ileal Histomorphometry Parameters

The ileal histomorphometry parameters of broiler chickens in response to treatment with thyme powder, prebiotic, probiotic, and their combinations are shown in Table 9. Ileal villus length was significantly higher in birds receiving T3 and T7 compared with birds in the T1, T2, T4, and T6 groups (*p* = 0.001). Birds in the T5 group did not differ significantly in villus length from T3, T7, or T6. Villus width was significantly higher in birds receiving T5 and T7 compared with birds in the T1, T2, and T3 groups (*p* = 0.001). The T4 and T6 groups did not differ significantly in villus width compared with the other treatment groups. In addition, villus surface area was significantly higher in birds receiving T5 and T7 compared with all other treatment groups (*p* = 0.001). Crypt depth was significantly higher in birds receiving T7 compared with birds in the T1, T3, and T6 groups (*p* = 0.003). The villus length-to-crypt depth ratio was significantly higher in birds receiving T3 and T6 compared with birds in the T1, T2, T4, and T7 groups (*p* = 0.006). Goblet cell counts were higher in birds receiving natural additive groups (T3–T7) compared with T1 and T2 groups (*p* = 0.001).

The contrast effect comparing natural additives with the controls (T1 or T2) affected all histomorphometry measurements (*p* < 0.05), except for crypt depth, which was not affected (*p* > 0.05). The main effects of thyme (T1 × T3), prebiotic (T1 × T4), and probiotic (T1 × T5) significantly influenced villus length and goblet cell counts, with the probiotic additionally affecting villus width, surface area, and the villus length-to-crypt depth ratio (*p* < 0.05). Significant two-way interactions were observed for T3 × T4 in villus length and width, T3 × T5 in villus width, surface area, and goblet cells, and T4 × T5 in villus length, surface area, and goblet cells (*p* < 0.05). Lastly, a significant three-way interaction (T3 × T4 × T5) was detected for villus length, villus width, and villus surface area (*p* < 0.05).

### 3.7. Ileal Tissue Occludin as an Intestinal Integrity Marker

**Figure 1:**
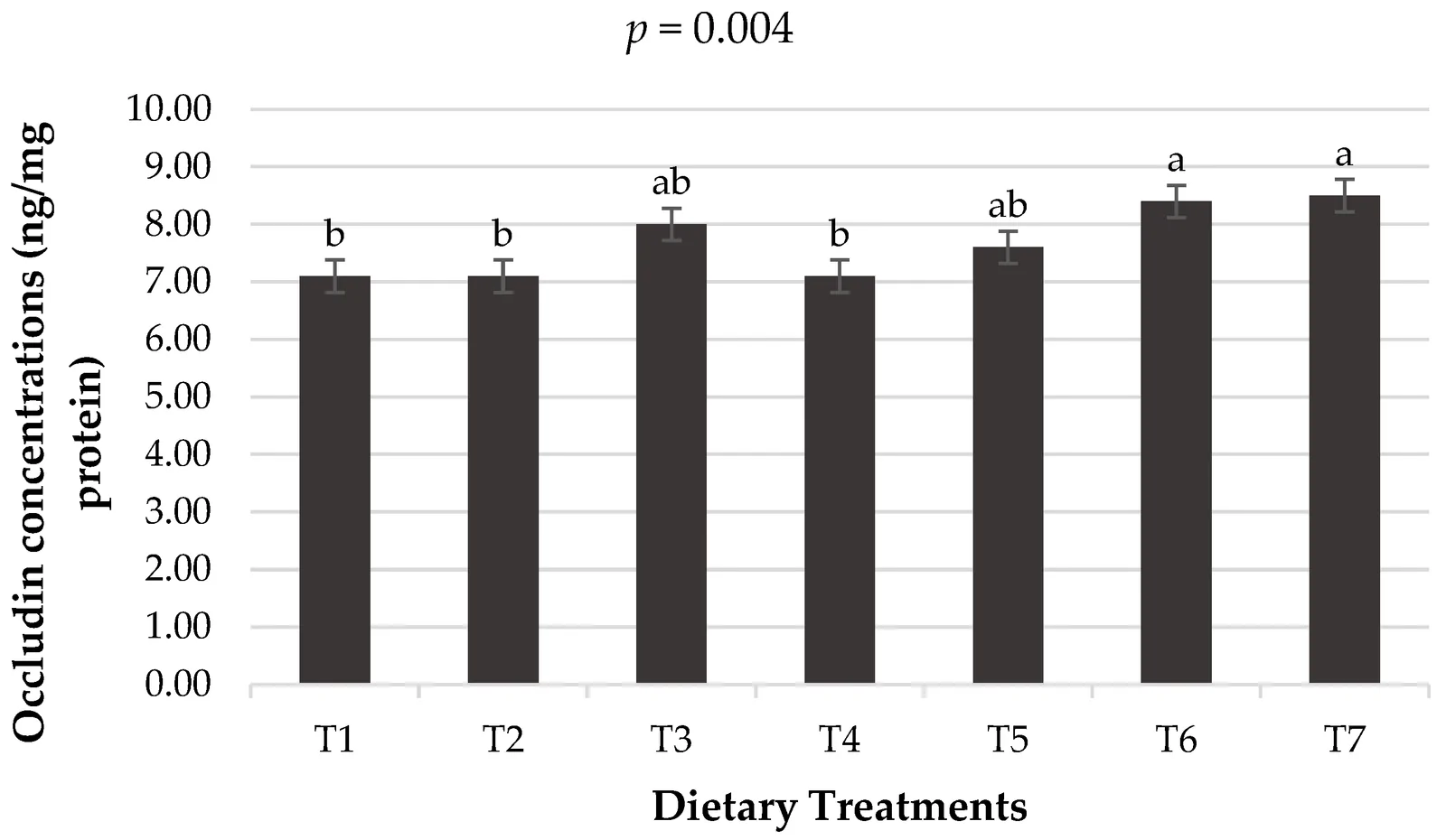
The ileal tissue occludin as an intestinal integrity marker of broiler chickens in response to treatment groups at 28 d. ^a–b^ Within each column, means bearing different superscripts differ significantly (Tukey’s test, *p* < 0.05; n = 13 birds per treatment). Treatment groups: T1 = basal diet; T2 = basal diet + 0.5 g/kg antibiotic reference; T3 = basal diet + 1.0 g/kg thyme powder; T4 = basal diet + 0.75 g/kg prebiotic; T5 = basal diet + 0.50 g/kg probiotic; T6 = basal diet + 0.75 g/kg prebiotic + 1.0 g/kg thyme; T7 = basal diet + 0.50 g/kg probiotic + 1.0 g/kg thyme. Data are presented as mean ± standard error of the mean (SEM).

The ileal tissue occludin concentration, as an intestinal integrity marker in broiler chickens in response to treatment with thyme powder, prebiotic, probiotic, and their combinations, is shown in Figure 1. Occludin concentration was significantly affected by the treatment groups (*p* = 0.004). Birds receiving thyme + prebiotic (T6) and thyme + probiotic (T7) showed significantly higher ileal occludin concentrations compared with birds in the T1, T2, and T4 groups (*p* < 0.05). Birds in the T3 and T5 groups did not show significantly different occludin concentrations compared with the other treatment groups.

## 4. Discussion

Optimizing productive performance over a compact 28-day commercial production cycle is a critical metric for evaluating alternative additives in modern poultry formulations. The uniformity of initial body weights across all experimental groups confirms robust randomization prior to the dietary intervention. The absence of significant treatment effects on growth performance and feed conversion ratio during the starter phase suggests that young chicks may have an immature digestive tract and developing microflora that limit their ability to immediately exploit the physiological benefits of phytogenic and biotic additives [30]. This delayed response indicates that the growth-promoting pathways of natural feed additives become fully operational as the intestinal tract matures and metabolic demands accelerate during the grower period [12,31].

During the grower and cumulative phases, dietary supplementation with thyme powder, prebiotics, probiotics, and the thyme–probiotic combination increased final body weight and cumulative weight gain compared with the unsupplemented control. The growth improvements observed in individual additive groups confirm their efficacy as standalone alternatives to AGPs. Thyme powder exerts digestive-stimulatory effects through its principal bioactive constituents, thymol and carvacrol [32], which were delivered at diet levels of 38.25 mg/kg and 1.13 mg/kg, respectively (yielding approximately 1.38 mg thymol and 0.041 mg carvacrol/kg BW/day during the grower period). These phenolic compounds disrupt bacterial cell membrane integrity, reduce enteric pathogen burden, and stimulate endogenous digestive enzyme secretion, thereby enhancing nutrient hydrolysis without altering voluntary feed intake [9,14,15,33,34]. Similarly, *Bacillus licheniformis* competitively excludes enteric pathogens, secretes antimicrobial bacteriocins against *Clostridium* spp. and *Salmonella* spp., and enhances proximal intestinal enzymatic activity [35,36]. The MOS/β-glucan prebiotic likely acted via mannose receptor-mediated pathogen exclusion and β-glucan-mediated immunomodulation, freeing metabolic energy for productive growth [37].

Regarding growth performance, birds receiving individual or combined additives showed statistically equivalent improvements in final body weight, cumulative weight gain, and feed conversion ratio. Although the thyme + probiotic group displayed numerically higher cumulative feed conversion ratio and final body weight, this numerical trend must not be overinterpreted as statistical synergy or additive interaction. Growth performance metrics showed no statistically significant two-way (thyme × prebiotic, thyme × probiotic, and prebiotic × probiotic) or three-way (thyme × prebiotic × probiotic) interaction terms. Consequently, performance gains reflect independent, non-synergistic contributions from individual additives—specifically, monoterpene-induced stimulation of endogenous digestive enzymes [14,34], MOS/β-glucan receptor blocking [37], and *Bacillus licheniformis*-mediated competitive exclusion [35,36].

The most compelling evidence of a synergistic interaction was the significant elevation of ileal occludin concentration in the thyme + prebiotic and thyme + probiotic groups—an effect not observed in any single-additive group. At the molecular level, tight junction protein assembly, including occludin, is tightly regulated by the TLR4/MyD88/NF-κB signaling cascade. Pathogen-associated molecular patterns (PAMPs), such as lipopolysaccharides, trigger host TLR4 dimerization, activating the MyD88-dependent pathway that leads to IκB kinase phosphorylation and nuclear translocation of NF-κB. Active NF-κB translocates to the nucleus to drive pro-inflammatory cytokine expression (e.g., TNF-α, IL-1*β*) and directly downregulates occludin gene transcription while inducing myosin light chain kinase (MLCK)-mediated tight junction disassembly [38]. The combination additives disrupt this pathological cascade through a dual-action pathway: thyme phenolics directly scavenge reactive oxygen species (ROS) and suppress TLR4 activation, attenuating upstream NF-κB signaling [39]. Biotic components supply SCFAs, such as butyrate and acetate, which bind enterocyte G-protein coupled receptors (GPR41/GPR43) and inhibit histone deacetylases (HDACs). This activation engages the PI3K/Akt and MAPK/ERK signaling axes, promoting enterocyte survival, upregulating MUC2 transcription, and restoring occludin localization at the apical junctional complex [40,41].

Because single additives target only isolated nodes within this regulatory network, individual supplementation produced non-significant changes in occludin levels. In contrast, combined supplementation reduced the upstream PAMP/TLR4 inflammatory burden while supplying luminal SCFA signaling molecules, resulting in significant upregulation of occludin expression. Although elevated occludin levels and improved villus architecture strongly suggest structural reinforcement of the mucosal surface, these parameters serve as structural proxies; direct functional physiological capacity (e.g., transepithelial electrical resistance or macromolecular flux) requires future ex vivo permeability validation [42].

Micromorphological parameters further demonstrated structural additive responses. The combination of thyme and probiotic produced significant additive effects on ileal villus surface area by combining the increased villus length induced by thyme with the lateral villus expansion (villus width) promoted by *Bacillus licheniformis*. This expansion was accompanied by increased crypt depth in the thyme + probiotic group, reflecting heightened enterocyte proliferation necessary to maintain a rapidly expanding absorptive surface, rather than mucosal pathology [43,44]. In contrast, the thyme alone and thyme + prebiotic groups exhibited higher villus length-to-crypt depth ratios, suggesting highly efficient mucosal architecture with lower baseline cell turnover costs.

Systemic antioxidant capacity and microbial profiling provided additional evidence of complementary interactions. The significant elevation in serum TAC and suppression of MDA across natural additive groups reflect the direct free-radical scavenging and metal-chelating activities of thyme phenolics [9], paired with probiotic-driven production of antioxidant metabolites and reduction in gut-derived ROS generation [45]. The limited direct antioxidant effect in the prebiotic-alone group underscores that MOS/β-glucan benefits operate indirectly via SCFA-mediated pathways rather than direct radical scavenging [46].

In the cecal microenvironment, the thyme + probiotic group maintained elevated total SCFA production by combining the strong propionic acid generation of *Bacillus licheniformis* with acetic acid fermentation stimulated by microbial shifts [47,48]. This fermentative landscape aligned with significant shifts in microbial populations: thyme selectively suppressed Gram-negative *Escherichia coli* through membrane destabilization and DNA gyrase inhibition [42], while favoring *Lactobacillus* and *Bifidobacterium* spp., which exhibit greater tolerance to phenolic compounds and thrive under SCFA-induced luminal acidification [22,49].

Unexpectedly, cytokine profiling revealed non-linear responses across the combined groups. Serum IL-6 was elevated in the thyme and probiotic groups, functioning as a pleiotropic signal that supports immune surveillance rather than systemic inflammation [50,51,52], whereas the thyme + prebiotic group exhibited a transient elevation in TNF-α. Rather than indicating systemic pathology, this response likely reflects localized mucosal immune priming driven by the interplay between prebiotic pattern-recognition receptor binding (via β-glucans) and phytogenic immune activation [53,54].

A central finding of this study is that the antibiotic reference group consistently failed to match the performance of all-natural additive formulations across critical intestinal health indices, proving significantly inferior in goblet cell density, butyric acid concentration, total SCFA production, and ileal occludin expression. The mechanistic explanation for this antibiotic-induced deficit lies in the non-selective antimicrobial activity of conventional AGPs. Broad-spectrum antibiotics deplete commensal anaerobic fermenters, particularly butyrate-producing Firmicutes clusters (e.g., *Faecalibacterium prausnitzii* and *Ruminococcus* spp.). This depletion creates an enteric “butyrate deficit,” depriving colonocytes and ileal enterocytes of their primary energy substrate [55]. Because intracellular butyrate signaling via GPR41/43 and HDAC inhibition is required to stimulate goblet cell differentiation and upregulate MUC2 and tight junction gene transcription [43,56], AGP-treated birds exhibited lower goblet cell counts, reduced mucosal gel layer thickness, and impaired occludin expression. Furthermore, the suppression of commensal microbial fermentation by AGPs lowers total luminal SCFA concentration, raising intestinal pH and diminishing the competitive exclusion of opportunistic pathogens. In contrast, natural additive strategies—whether applied individually or in combination—preserve and enhance the functional fermentative niche of the microbiota. By stimulating endogenous SCFA production, selectively inhibiting pathobionts via targeted membrane disruption (thyme) or competitive exclusion (*Bacillus licheniformis* and MOS), and activating enterocyte PI3K/Akt survival pathways, natural feed additives maintain dynamic mucosal homeostasis and structural epithelial integrity.

While the current findings demonstrate clear physiological benefits of combining wild thyme powder with a probiotic, several experimental limitations must be acknowledged. First, the plant material evaluated originated from a single harvest batch in Ibb City, Yemen; geographic, seasonal, and chemotypic variation in monoterpene profiles (38.25 mg/g thymol) may influence batch-to-batch reproducibility. Second, additives were tested at single, fixed inclusion rates (1.0 g/kg thyme, 0.75 g/kg prebiotic, 0.50 g/kg probiotic), precluding the determination of optimal dose–response kinetics or minimum effective dosages. Third, trials were conducted in healthy, non-challenged broilers under controlled experimental conditions; thus, responses under field stress or acute pathogen challenge (e.g., *Eimeria* spp. or *Clostridium perfringens*) remain to be established. Fourth, regarding biomarker selection, systemic oxidative status was evaluated using TAC and MDA as operational indices of overall non-enzymatic radical-scavenging capacity and lipid peroxidation damage, respectively, without measuring specific primary enzymatic antioxidants such as superoxide dismutase, catalase, or glutathione peroxidase. Similarly, intestinal physical integrity was assessed solely through occludin concentration as a representative key transmembrane tight junction protein, excluding complementary junctional components such as Zonula Occludens-1 and claudins. Finally, biological sampling was cross-sectional at day 28, preventing the tracking of dynamic microbiota successions across earlier rearing phases. Future studies should employ multidose factorial designs, longitudinal sampling, and enteric infection models to further refine these dietary interventions.

## 5. Conclusions

Under the specific conditions of this trial, dietary supplementation with *Thymus vulgaris* powder (1.0 g/kg), a MOS/β-glucan prebiotic (0.75 g/kg), and a *Bacillus licheniformis* probiotic (0.50 g/kg) improved growth performance, antioxidant capacity, cecal metabolite production, and ileal tight junction integrity in healthy broiler chickens. Birds receiving thyme together with the probiotic showed consistent numerical advantages in final body weight and cumulative feed conversion ratio, along with significant enhancements in villus surface area, total SCFA concentration, and ileal occludin protein expression. In addition, the occludin results provide the most direct evidence of combined effects between the phytogenic and biotic components, achievable only through their co-supplementation.

The antibiotic reference showed lower values than the natural additive groups for several intestinal health outcomes and was significantly inferior for butyric acid production and goblet cell differentiation, highlighting the potential value of these antibiotic-free alternatives in broiler nutrition. However, these outcomes reflect a single plant harvest, single inclusion levels, non-challenged flock conditions, and a cross-sectional sampling endpoint. While the thyme + probiotic combination shows promising potential for supporting broiler gut health under experimental conditions, these findings should not be interpreted as a definitive indication that natural additives can fully replace antibiotic growth promoters in commercial settings. Extrapolation to commercial production systems requires further validation across diverse plant batches, varying inclusion doses, and disease-challenge environments. Future research should investigate dose–response relationships for each component and evaluate performance under pathogen-challenge models to further establish the translational applicability and AGP replacement potential of these findings.

## Figures and Tables

**Table 1:** Ingredients and nutrient composition of basal diets during starter and grower stages.

Ingredients (% as Feed)	Dietary Stages	
	Starter (0–14 d)	Grower (15–28 d)
Corn	53.28	53.61
Soybean Meal (48% CP)	38.32	37.52
Soy Oil	3.57	4.94
Monocalcium Phosphate	1.68	1.40
Limestone	1.60	1.39
DL-Methionine	0.41	0.33
L-Lysine HCL	0.30	0.14
L-Threonine	0.18	0.10
Common Salt	0.43	0.38
Vitamin Premix ^a^	0.10	0.10
Mineral Premix ^b^	0.10	0.10
Choline Cl-60%	0.04	0.00
Total	100.00	100.00
**Calculated Nutrients (% as dry matter)**		
Dry Matter	88.97	89.02
Metabolizable Energy (kcal/kg)	3000.00	3100.46
Crude Protein	22.64	22.06
Ether Extract	6.30	7.66
Crude Fiber	2.54	2.51
Calcium	1.00	0.87
Avail. Phosphorus	0.50	0.43
Sodium	0.18	0.16
Chlorine	0.29	0.26
Lysine	1.32	1.18
Methionine	0.70	0.62
Total Sulfur Amino Acids	0.98	0.90
Threonine	0.88	0.79

^a^ Vitamin premix composition per kg: A, 2,350,000 IU; D, 1,100,000 IU; E, 16,500 IU; K, 800 mg; B1, 750 mg; B2, 1650 mg; B6, 1100 mg. ^b^ Mineral premix composition per kg: Co, 85 mg; Cu, 1800 mg; I, 450 mg; Fe, 1250 mg; Mn, 18,000 mg; Se, 60 mg; Zn, 14,000 mg.

**Table 2:** Quantitative characterization and GC-MS/HPLC profile of active compounds in wild thyme (*Thymus vulgaris* L.) powder.

RT (min)	Compound/Parameter	Relative Concentration (%)	Concentration in Powder (mg/g DM)
14.657	Thymol	72.92 ± 1.45	38.25 ± 0.95
16.224	Carvacrol	2.16 ± 0.12	1.13 ± 0.08
8.412	*p*-Cymene	4.85 ± 0.22	2.54 ± 0.11
9.105	Gamma-Terpinene	3.12 ± 0.18	1.63 ± 0.09
9.896	Undecane	7.21 ± 0.35	-
27.096	*n*-Hexadecanoic acid	8.09 ± 0.41	-
-	Total Phenolics	-	38.45 ± 1.20 mg GAE/g
-	Total Flavonoids	-	14.12 ± 0.85 mg QE/g

Values are presented as mean ± standard deviation (n = 3). DM = dry matter; GAE = gallic acid equivalents; QE = quercetin equivalents.

**Table 4:** The productive performance of broiler chickens in response to treatment groups over the full 28 d trial.

Treatment Groups ^1^	Body Weight (g)			Weight Gain (g)			Feed Intake (g)			Feed Conversion Ratio (g/g)		
	0 d	14 d	28 d	0–14 d	15–28 d	0–28 d	0–14 d	15–28 d	0–28 d	0–14 d	15–28 d	0–28 d
T1	43.02	483	1536 ^b^	440	1053 ^b^	1493 ^b^	517	1596	2113	1.18	1.52 ^a^	1.42 ^a^
T2	43.03	486	1602 ^ab^	443	1115 ^ab^	1559 ^ab^	524	1624	2148	1.18	1.46 ^ab^	1.38 ^ab^
T3	43.01	500	1649 ^a^	457	1150 ^a^	1606 ^a^	520	1624	2144	1.14	1.41 ^ab^	1.34 ^b^
T4	43.02	501	1652 ^a^	458	1152 ^a^	1609 ^a^	537	1643	2180	1.17	1.43 ^ab^	1.36 ^ab^
T5	43.03	500	1633 ^a^	457	1133 ^a^	1590 ^a^	526	1609	2135	1.15	1.42 ^ab^	1.34 ^b^
T6	43.03	497	1613 ^ab^	454	1116 ^ab^	1570 ^ab^	533	1603	2136	1.18	1.44 ^ab^	1.36 ^ab^
T7	43.03	508	1668 ^a^	465	1160 ^a^	1625 ^a^	523	1609	2132	1.13	1.39 ^b^	1.31 ^b^
**SEM ^2^**	0.048	5.780	18.98	5.774	17.55	18.96	8.042	15.20	17.15	0.022	0.023	0.015
**Source of variance (** ***p*** **-value)**												
Treatments	0.999	0.054	0.003	0.055	0.001	0.003	0.616	0.366	0.226	0.501	0.014	0.001
T1 vs. T3–T7	0.931	0.006	0.001	0.006	0.001	0.001	0.230	0.198	0.091	0.363	0.003	0.001
T2 vs. T3–T7	0.918	0.025	0.043	0.026	0.173	0.042	0.711	0.710	0.873	0.258	0.047	0.042
T1 × T3	0.884	0.042	0.001	0.041	0.004	0.001	0.783	0.200	0.208	0.259	0.003	0.009
T1 × T4	1.000	0.034	0.001	0.034	0.003	0.001	0.092	0.032	0.084	0.930	0.009	0.009
T1 × T5	0.884	0.042	0.008	0.041	0.002	0.008	0.447	0.553	0.372	0.485	0.005	0.002
T3 × T4	0.884	0.930	0.911	0.930	0.922	0.915	0.156	0.364	0.143	0.297	0.729	0.412
T3 × T5	0.770	1.000	0.540	1.000	0.501	0.536	0.626	0.485	0.708	0.662	0.896	0.751
T4 × T5	0.884	0.930	0.470	0.930	0.441	0.469	0.345	0.112	0.068	0.541	0.828	0.613
T3 × T4 × T5	0.800	0.960	0.771	0.960	0.738	0.767	0.270	0.902	0.524	0.393	0.783	0.511

^a–b^ Within each column, means bearing different superscripts differ significantly (Tukey’s test, *p* < 0.05; n = 13 cages per treatment). ^1^ T1 = basal diet; T2 = basal diet + 0.5 g/kg antibiotic reference; T3 = basal diet + 1.0 g/kg thyme powder; T4 = basal diet + 0.75 g/kg prebiotic; T5 = basal diet + 0.50 g/kg probiotic; T6 = basal diet + 0.75 g/kg prebiotic + 1.0 g/kg thyme; T7 = basal diet + 0.50 g/kg probiotic + 1.0 g/kg thyme. ^2^ SEM = standard error of the mean.

**Table 5:** The serum oxidative status biomarkers of broiler chickens in response to treatment groups at 28 d.

Treatment Groups ^2^	Oxidative Status Biomarkers ^1^	
	TAC (µmol/mL)	MDA (nmol/mL)
T1	15.97 ^b^	3.86 ^a^
T2	15.94 ^b^	3.58 ^a^
T3	18.31 ^a^	2.38 ^b^
T4	16.20 ^b^	3.79 ^a^
T5	17.23 ^ab^	2.49 ^b^
T6	16.82 ^b^	2.45 ^b^
T7	18.04 ^a^	2.13 ^b^
**SEM ^3^**	0.415	0.171
**Source of variance (** ***p*** **-value)**		
Treatments	0.004	0.001
T1 vs. T3–T7	0.005	0.001
T2 vs. T3–T7	0.004	0.001
T1 × T3	0.003	0.001
T1 × T4	0.700	0.769
T1 × T5	0.039	0.001
T3 × T4	0.001	0.001
T3 × T5	0.074	0.638
T4 × T5	0.088	0.001
T3 × T4 × T5	0.003	0.009

^a–b^ Within each column, means bearing different superscripts differ significantly (Tukey’s test, *p* < 0.05; n = 13 birds per treatment). ^1^ TAC = total antioxidant capacity and MDA = malondialdehyde. ^2^ T1 = basal diet; T2 = basal diet + 0.5 g/kg antibiotic reference; T3 = basal diet + 1.0 g/kg thyme powder; T4 = basal diet + 0.75 g/kg prebiotic; T5 = basal diet + 0.50 g/kg probiotic; T6 = basal diet + 0.75 g/kg prebiotic + 1.0 g/kg thyme; T7 = basal diet + 0.50 g/kg probiotic + 1.0 g/kg thyme. ^3^ SEM = standard error of the mean.

**Table 6:** The immune response markers of broiler chickens in response to treatment groups at 28 d.

Treatment Groups ^2^	Immune Response Markers (ng/L) ^1^		
	IL-6	TNF-α	IFN-γ
T1	340 ^b^	299 ^b^	275 ^b^
T2	329 ^b^	299 ^b^	272 ^b^
T3	404 ^a^	229 ^c^	397 ^a^
T4	374 ^b^	272 ^c^	300 ^b^
T5	402 ^a^	310 ^ab^	362 ^a^
T6	352 ^b^	359 ^a^	380 ^a^
T7	417 ^a^	323 ^ab^	409 ^a^
**SEM ^3^**	21.14	17.43	14.79
**Source of variance (** ***p*** **-value)**			
Treatments	0.021	0.001	0.001
T1 vs. T3–T7	0.039	0.979	0.001
T2 vs. T3–T7	0.012	0.999	0.001
T1 × T3	0.033	0.005	0.001
T1 × T4	0.255	0.260	0.207
T1 × T5	0.040	0.643	0.001
T3 × T4	0.272	0.063	0.001
T3 × T5	0.935	0.001	0.073
T4 × T5	0.311	0.100	0.003
T3 × T4 × T5	0.497	0.003	0.004

^a–c^ Within each column, means bearing different superscripts differ significantly (Tukey’s test, *p* < 0.05; n = 13 birds per treatment). ^1^ IL-6 = interleukin-6, TNF-α = tumor necrosis factor-α, and IFN-γ = interferon-γ. ^2^ T1 = basal diet; T2 = basal diet + 0.5 g/kg antibiotic reference; T3 = basal diet + 1.0 g/kg thyme powder; T4 = basal diet + 0.75 g/kg prebiotic; T5 = basal diet + 0.50 g/kg probiotic; T6 = basal diet + 0.75 g/kg prebiotic + 1.0 g/kg thyme; T7 = basal diet + 0.50 g/kg probiotic + 1.0 g/kg thyme. ^3^ SEM = standard error of the mean.

**Table 7:** The cecal short-chain fatty acids (SCFAs) concentrations of broiler chickens in response to treatment groups at 28 d.

Treatment Groups ^1^	The SCFAs Concentrations (mg/g)			
	Acetic Acid	Propionic Acid	Butyric Acid	Total SCFA
T1	23.81 ^b^	2.12 ^d^	4.66 ^b^	30.59 ^c^
T2	24.00 ^b^	3.49 ^bc^	3.15 ^c^	30.65 ^c^
T3	24.63 ^b^	4.07 ^b^	4.86 ^b^	33.56 ^bc^
T4	30.76 ^a^	2.21 ^d^	4.89 ^b^	37.86 ^b^
T5	33.04 ^a^	5.48 ^a^	6.35 ^a^	44.87 ^a^
T6	30.45 ^a^	2.40 ^cd^	5.28 ^ab^	38.13 ^b^
T7	35.07 ^a^	5.73 ^a^	4.43 ^b^	45.23 ^a^
**SEM ^2^**	1.251	0.240	0.334	1.250
**Source of variance (** ***p*** **-value)**				
Treatments	0.001	0.001	0.001	0.001
T1 vs. T3–T7	0.001	0.001	0.184	0.001
T2 vs. T3–T7	0.001	0.074	0.001	0.001
T1 × T3	0.648	0.001	0.681	0.107
T1 × T4	0.001	0.793	0.635	0.005
T1 × T5	0.001	0.001	0.001	0.001
T3 × T4	0.002	0.001	0.950	0.024
T3 × T5	0.001	0.004	0.004	0.001
T4 × T5	0.212	0.001	0.006	0.007
T3 × T4 × T5	0.001	0.460	0.077	0.001

^a–d^ Within each column, means bearing different superscripts differ significantly (Tukey’s test, *p* < 0.05; n = 13 birds per treatment). ^1^ T1 = basal diet; T2 = basal diet + 0.5 g/kg antibiotic reference; T3 = basal diet + 1.0 g/kg thyme powder; T4 = basal diet + 0.75 g/kg prebiotic; T5 = basal diet + 0.50 g/kg probiotic; T6 = basal diet + 0.75 g/kg prebiotic + 1.0 g/kg thyme; T7 = basal diet + 0.50 g/kg probiotic + 1.0 g/kg thyme. ^2^ SEM = standard error of the mean.

**Table 8:** The quantification of the selected cecal microbiota of broiler chickens in response to treatment groups at 28 d.

Treatment Groups ^1^	Cecal Microbiota (log_10_ Gene Copy Numbers per Gram of Cecal Digesta)				
	*Lactobacillus* spp.	*Bifidobacteria* spp.	*Bacteroides* spp.	*Clostridium* spp.	*Escherichia coli*
T1	3.43 ^c^	3.45 ^b^	6.42	2.54 ^a^	4.24 ^a^
T2	3.66 ^c^	3.39 ^b^	6.01	2.00 ^ab^	3.23 ^ab^
T3	4.31 ^b^	3.65 ^ab^	7.51	1.79 ^b^	2.90 ^b^
T4	4.93 ^a^	3.65 ^ab^	6.91	1.89 ^b^	3.81 ^ab^
T5	4.52 ^a^	4.12 ^ab^	6.42	1.81 ^b^	3.63 ^ab^
T6	4.52 ^a^	3.89 ^ab^	7.17	1.81 ^b^	3.61 ^ab^
T7	4.65 ^a^	4.43 ^a^	6.72	1.95 ^b^	2.93 ^b^
**SEM ^2^**	0.167	0.180	0.352	0.130	0.231
**Source of variance (** ***p*** **-value)**					
Treatments	0.001	0.003	0.087	0.004	0.003
T1 vs. T3–T7	0.001	0.019	0.183	0.001	0.002
T2 vs. T3–T7	0.001	0.008	0.021	0.309	0.588
T1 × T3	0.009	0.467	0.037	0.004	0.004
T1 × T4	0.001	0.467	0.331	0.001	0.202
T1 × T5	0.001	0.014	0.996	0.005	0.074
T3 × T4	0.013	1.000	0.239	0.615	0.010
T3 × T5	0.370	0.069	0.036	0.915	0.035
T4 × T5	0.096	0.069	0.329	0.692	0.587
T3 × T4 × T5	0.050	0.285	0.060	0.725	0.007

^a–c^ Within each column, means bearing different superscripts differ significantly (Tukey’s test, *p* < 0.05; n = 13 birds per treatment). ^1^ T1 = basal diet; T2 = basal diet + 0.5 g/kg antibiotic reference; T3 = basal diet + 1.0 g/kg thyme powder; T4 = basal diet + 0.75 g/kg prebiotic; T5 = basal diet + 0.50 g/kg probiotic; T6 = basal diet + 0.75 g/kg prebiotic + 1.0 g/kg thyme; T7 = basal diet + 0.50 g/kg probiotic + 1.0 g/kg thyme. ^2^ SEM = standard error of the mean.

**Table 9:** The ileal histomorphometry of broiler chickens in response to treatment groups at 28 d.

Treatment Groups ^1^	Ileal Histomorphometry					
	Villus Length (μm)	Villus Width (μm)	Villus Surface Area (mm^2^)	Crypt Depth (μm)	Villus Length/Crypt Depth	Goblet Cells (NO.)
T1	465.9 ^c^	110.7 ^b^	0.16 ^b^	60.6 ^b^	7.79 ^b^	92.0 ^b^
T2	483.8 ^c^	112.0 ^b^	0.17 ^b^	63.4 ^ab^	7.74 ^b^	81.4 ^c^
T3	548.5 ^a^	103.4 ^b^	0.18 ^b^	60.7 ^b^	9.12 ^a^	120.1 ^a^
T4	498.7 ^b^	120.2 ^ab^	0.19 ^b^	61.7 ^ab^	8.30 ^b^	120.7 ^a^
T5	537.7 ^ab^	131.6 ^a^	0.22 ^a^	62.5 ^ab^	8.75 ^ab^	127.7 ^a^
T6	509.5 ^b^	115.5 ^ab^	0.18 ^b^	57.7 ^b^	9.00 ^a^	127.8 ^a^
T7	544.7 ^a^	132.6 ^a^	0.23 ^a^	70.5 ^a^	7.83 ^b^	127.7 ^a^
**SEM ^2^**	10.72	4.291	0.007	2.106	0.333	2.181
**Source of variance (** ***p*** **-value)**						
Treatments	0.001	0.001	0.001	0.003	0.006	0.001
T1 vs. T3–T7	0.001	0.036	0.001	0.394	0.029	0.001
T2 vs. T3–T7	0.003	0.039	0.004	0.749	0.020	0.001
T1 × T3	0.001	0.233	0.151	0.993	0.005	0.001
T1 × T4	0.033	0.121	0.016	0.730	0.282	0.001
T1 × T5	0.001	0.009	0.001	0.526	0.046	0.001
T3 × T4	0.001	0.006	0.326	0.736	0.086	0.846
T3 × T5	0.479	0.001	0.001	0.531	0.430	0.014
T4 × T5	0.011	0.063	0.009	0.772	0.349	0.024
T3 × T4 × T5	0.023	0.001	0.002	0.578	0.147	0.125

^a–c^ Within each column, means bearing different superscripts differ significantly (Tukey’s test, *p* < 0.05; n = 13 birds per treatment). ^1^ T1 = basal diet; T2 = basal diet + 0.5 g/kg antibiotic reference; T3 = basal diet + 1.0 g/kg thyme powder; T4 = basal diet + 0.75 g/kg prebiotic; T5 = basal diet + 0.50 g/kg probiotic; T6 = basal diet + 0.75 g/kg prebiotic + 1.0 g/kg thyme; T7 = basal diet + 0.50 g/kg probiotic + 1.0 g/kg thyme. ^2^ SEM = standard error of the mean.

## Data Availability

The original contributions presented in this study are included in the article. Further inquiries can be directed to the corresponding author.
